# Bimanual Fundamentals: Validation of a New Curriculum for Virtual Reality Training of Laparoscopic Skills

**DOI:** 10.1177/1553350620953030

**Published:** 2020-08-29

**Authors:** Martijn P. H. van Ginkel, Marlies P. Schijven, Wilhelmina M. U. van Grevenstein, Henk W. R. Schreuder

**Affiliations:** 1 Department of Obstetrics and Gynecology, 8124 University Medical Center Utrecht, the Netherlands; 2 Department of Surgery, University of Amsterdam, the Netherlands; 3 Department of Surgery, 8124 University Medical Center Utrecht, the Netherlands; 4 Department of Gynecologic Oncology, Cancer Center, 8124 University Medical Center Utrecht, the Netherlands

**Keywords:** laparoscopy, simulation, virtual reality, validity, minimally invasive surgery, training

## Abstract

*Background.* To determine face and construct validity for the new Bimanual Fundamentals curriculum for the Simendo^®^ Virtual Reality Laparoscopy Simulator and prove its efficiency as a training and objective assessment tool for surgical resident’s advanced psychomotor skills. *Methods.* 49 participants were recruited: 17 medical students (novices), 15 residents (intermediates), and 17 medical specialists (experts) in the field of gynecology, general surgery, and urology in 3 tertiary medical centers in the Netherlands. All participants performed the 5 exercises of the curriculum for 3 consecutive times on the simulator. Intermediates and experts filled in a questionnaire afterward, regarding the reality of the simulator and training goals of each exercise. Statistical analysis of performance was performed between novices, intermediates, and experts. Parameters such as task time, collisions/displacements, and path length right and left were compared between groups. Additionally, a total performance score was calculated for each participant. *Results.* Face validity scores regarding realism and training goals were overall positive (median scores of 4 on a 5-point Likert scale). Participants felt that the curriculum was a useful addition to the previous curricula and the used simulator would fit in their residency programs. Construct validity results showed significant differences on the great majority of measured parameters between groups. The simulator is able to differentiate between performers with different levels of laparoscopic experience. *Conclusions.* Face and construct validity for the new Bimanual Fundamental curriculum for the Simendo virtual reality simulator could be established. The curriculum is suitable to use in resident’s training programs to improve and maintain advanced psychomotor skills.

## Introduction

In laparoscopic surgery, surgeons need to use psychomotor abilities that substantially differ from those used in conventional surgery (eg, different eye-to-hand coordination, conversion of three-dimensional to two-dimensional images, altered tactile feedback, and the fulcrum effect). Before being able to perform laparoscopic surgery on patients, these psychomotor abilities need to be trained.^1,2^ However, the traditional training of residents, based on an apprenticeship-based model of teaching in the operating room (OR),^3^ can be time consuming,^4^ costly,^5^ and cause potential harm for patients.^6^ Moreover, in laparoscopy, residents often may only manipulate 1 or more fixed instruments—resulting in little opportunity to practice actual laparoscopic maneuvers during the operation.^7,8^

Therefore, alternative methods for training laparoscopy have been developed, such as box trainers, practicing on live animals or cadavers, and virtual reality (VR) simulation. VR offers great potential to improve and maintain technical and nontechnical skills outside the OR. It allows for a more flexible controlled environment without supervision, free of pressure of operating on patients, and without exposing patients to unnecessary risks.^9,10^ In the last decade, training using VR simulation has widely spread in surgical training curricula. Successful completion on VR simulators is nowadays often required for residents to perform laparoscopic surgery in real practice.^12,13^ The use of simulators for improving and maintaining laparoscopic skills is well supported by evidence.^9,11^ Skills acquired on a VR simulator are transferable to actual medical practice.^9,10,14-17^

The Simendo^®^ VR Simulator (Simendo B.V., Rotterdam, The Netherlands) is a laparoscopic VR simulator aimed at improving laparoscopic skills, for example, orientation, eye-to-hand coordination, precision of instrument handling, and (bi)manual movement in nonanatomic models. Previous studies have demonstrated face and construct validity for several basic and advanced exercises.^18,19^

Although the previous curriculum included advanced exercises, there is a need for an even higher level of complexity. Therefore, a new curriculum was developed, consisting of a variety of new exercises especially focused on ambidextrous skill development. Ambidextrous skill development requires a relatively high skill level. It provides a new challenge for residents who have succeeded the previous curricula. The purpose of this study was to determine face and construct validity for the newly developed set of exercises for training and assessment of advanced bimanual laparoscopic skills on this VR simulator.

## Materials and Methods

### Study Design

This prospective, multicenter, cohort study was conducted at the University Medical Center Utrecht, the Academic Medical Center Amsterdam, and the Erasmus University Medical Center Rotterdam at the gynecology, general surgery, and urology departments.

### Curriculum

**Figure 1.**
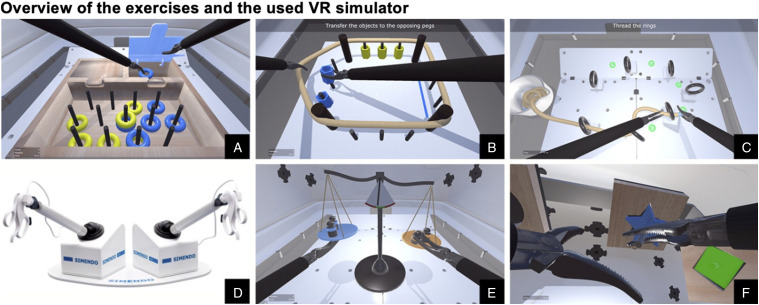
(A) Exercise “Sort the Rings,” (B) exercise “Stretch and Transfer,” (C) exercise “Ring and Rope,” (D) Simendo VR laparoscopy simulator, (E) exercise “Balance,” and (F) Exercise “Puzzle.” Images provided and copyrighted by Simendo®.

The new “Bimanual Fundamentals” curriculum was developed during the past years prior to this study, in cooperation with medical specialists, to achieve an appropriate level of difficulty and realism. It consists of 5 advanced exercises (Figure 1), especially focused on training and maintaining ambidextrous skills and cooperation between left and right instrument. The exercises are named “*Sort the Rings*” Supplemental Video 1, “*Stretch and Transfer*”*’* Supplemental Video 2, “*Ring and Rope*” Supplemental Video 3, “*Balance*” Supplemental Video 4, and “*Puzzle*” (Supplemental Video 5) and are stated in the ascending level of difficulty. The exercises, their training goals, and the measured parameters are described in Table 1.

**Table 1.** Overview of the 5 Exercises of the “*Bimanual Fundamentals*” Curriculum.

Name	Description	Training Goal	Measured Parameter	Performance Score Range
Sort the rings	Rings need to be placed in the boxes with the corresponding color. Boxes can only be opened with the same sided instrument	Bimanual dexterity and efficiency	Task time, collisions^a^, path length right, and path length left	4-12
Stretch and transfer	After looping the elastic cord around the pillars, pegs need to be transferred to the opposite site, without touching the elastic cord or each other	Bimanual dexterity	Task time, collisions^b^, path length right, and path length left	4-12
Ring and rope	A rope needs to be maneuvered through rings along a path	Precision and bimanual dexterity	Task time, collisions^c^, path length right, and path length left	4-12
Balance	Weights need to be put on a scale, while the scale must be kept in balance	Precision, bimanual dexterity, and stability	Task time, displacement^d^, path length right, path and length left	4-12
Puzzle	Puzzle pieces need to be placed in the correct spot	Precision and bimanual dexterity	Task time, path length right, and path length left	3-9

a A collision was registered when the instrument hit the edge of the ring-collecting bins.

b A collision was registered when a peg touched the elastic cord or another peg.

c A collision was registered when the instrument hit a ring with force.

d Displacement was registered when putting a weight on the scale, while the scale was not balanced.

### Recruitment of Participants

Participants were recruited on a voluntary basis from the participating centers. Participants consisted of medical students (years 4-6), residents (postgraduate years (PGY) 4-6 gynecology, urology, and general surgery), and medical specialists (gynecologists, surgeons, and urologists). Based on the status and laparoscopic experience, 3 groups were formed: novices (medical students), intermediates (residents), and experts (minimal invasive surgery specialists). To be considered an expert, the medical specialist must have had extended experience with selected laparoscopic procedures (at least 50 times for 2 of the procedures or more than 100 times for 1 of the procedures). For gynecologists, the selected procedures consisted of laparoscopic hysterectomy, laparoscopic oophorectomy, and laparoscopic pelvic lymphadenectomy. For general surgeons, the selected procedures consisted of laparoscopic cholecystectomy, Nissen fundoplication, laparoscopic colectomy, and laparoscopic bariatric procedures. For urologists, the laparoscopic prostatectomy and laparoscopic nephrectomy were chosen as selected procedures. All participants were asked about their prior experience with laparoscopic skills training. Experience with box trainers, VR trainers, and live animal training was estimated in hours. Participants’ demographics and laparoscopic theater experience (in hours) were also evaluated.

### Equipment

The Simendo^®^ VR simulator for laparoscopic skills training was used. This simulator consists of a software interface and 2 hardware instruments (Figure 1D) connected with 2 USB plugs to a laptop computer (Asus ROG Strix GL553VW, AsusTek Computer Inc. Taipei, Taiwan). The laptop contained an Intel^®^Core™ i5-6300HQ CPU (2.30 GHz, 8 GB RAM; Intel Corporation, Santa Clara, USA), with an NVIDIA GeForce GTX 960M graphics card, 15.4^″^ full HD LCD display, and Microsoft Windows 10 software (Microsoft Corporation, Redmond, USA).

### Face Validity

Face validity was defined as the degree of resemblance between the simulator and the laparoscopic procedure in real practice.^20,21^ To determine face validity, participants with experience in real practice were asked to complete a questionnaire immediately after completing the simulation procedure. The questionnaire contained 15 questions about the realism of the simulator, training capacities in general, and the suitability for training residents or surgeons. Additionally, questions were asked about the 5 exercises separately (6 questions per exercise). Questions were presented on a 5-point Likert scale.^22^ The last 2 questions regarded statements comparing the “Bimanual Fundamentals Curriculum” with the existing “Intermediate Curriculum” and concerning the implementation of the VR simulator in the current residency training programs. These could be answered with “agree,” “disagree,” or “no opinion.”

### Construct Validity

Construct validity was defined as the simulator’s ability to differentiate subjects with different levels of skills.^18^ Since this simulation addresses technical skills, it is expected that it differentiates between experienced and nonexperienced performers. In simulation validation studies, construct validity usually refers to the ability of the simulator to differentiate performance between surgical experts and novices.^23^ Construct validity is considered to be necessary before using a simulator in surgical training curricula and is preferable before using the simulator as a training tool.

To determine construct validity, participants completed 3 consecutive repetitions of each of the 5 exercises of the new curriculum. Before starting a new exercise, an instruction video was shown and the test supervisor gave a brief standardized explanation. The first run was meant to familiarize with the simulator. Verbal instructions were given by the test supervisor when necessary. The second and third runs were used for analysis. No verbal or other instructions were given during the second and third runs. For each exercise, the following parameters were documented and compared between the different groups: task time, collisions/displacements, and path length right and left. Task time was measured in seconds; it was determined as the time between retracting the instruments to start the run and completing the exercise. The number of collisions was obtained in exercises “Sort the Rings,” “Stretch and Transfer,” and “Ring and Rope.” Displacements were measured in the exercise “Balance” (Table 1). Collisions/displacements and path length (the distance covered by each instrument) were registered in arbitrary units.

To evaluate construct validity and get an objective score of all measured parameters combined, an overall performance score was calculated for each participant. For each parameter, in each exercise, quartile scores of the whole sample size were determined and used as cutoff points. The best 25% performers received 3 points and the worst 25% 1 point. Everyone in between (the middle 50%) received 2 points. Based on the number of measured parameters and exercises, the overall performance score ranged from 19 to 57 (Table 1).

### Use of Statistics

Data were analyzed with the Statistical Package for the Social Sciences version 22 (SPSS, Chicago, USA). We used descriptive statistics to describe characteristics of participants and groups. We differentiated between groups with the use of the nonparametric Kruskal–Wallis. Comparison between performances of groups was undertaken with the use of the Mann–Whitney U test. To determine the minimum sample size, a power analysis was performed. A total sample of 45 participants (3 groups of 15 participants) achieved a power of .80 with the one-way independent ANOVA calculation and an estimated effect size of .5. A level of *P* < .05 was considered statistically significant. Values are presented as medians with interquartile ranges unless stated otherwise.

### Ethics

The study was reviewed and approved by the Dutch Society for Medical Education (NVMO) Ethical Review Board (number 1033 and date April 23, 2018).

## Results

A total of 49 participants were enrolled in this study. The novice group consisted of 17 medical students aged 21-27 years. The intermediate group consisted of 15 PGY 4-6 residents, mostly active in gynecology (66.7%), 20% in urology, and 13.3% in general surgery. The expert group consisted of 17 minimally invasive surgeons, 58.8% were active in gynecology, 35.3% in general surgery, and 5.9% in urology. Participant characteristics including gender and hand dominance are summarized in Table 2. All 49 participants completed the 5 exercises 3 consecutive times in sequence and filled in the questionnaire afterward.

**Table 2.** Characteristics of the Participants Divided Into Groups.

Total (n = 49)	Group 1: Novice n = 17	Group 2: Intermediate n = 15	Group 3: Expert n = 17
Sex			
Male (%)	n = 8 (47.1)	n = 8 (53.3)	n = 13 (76.5)
Female (%)	n = 9 (52.9)	n = 7 (46.7)	n = 4 (23.5)
	23.4 (1.8)	33.7 (2.0)	46.9 (8.0)
Mean age (SD)			
Years^a^ (SD)	4.9 (1.0)	4.3 (.8)	12.2 (7.2)
Dominant hand			
Right (%)	n = 15 (88.2)	n = 13 (86.7)	n = 16 (94.1)
Left (%)	n = 2 (11.8)	n = 2 (13.3)	n = 1 (5.9)
Criteria	Med students years 4-6	Residents PGY 4-6	Gynecologists, general surgeons, or urologists
	No prior laparoscopic	No extended laparoscopic experience^b^	Extended laparoscopic experience^b^
Distribution within group			
Specialism			
Gynecology	N/A	n = 10 (66.7)	n = 10 (58.8)
General surgery	N/A	n = 2 (13.3)	n = 6 (35.3)
Urology	N/A	n = 3 (20.0)	n = 1 (5.9)

Abbreviation: PGY = postgraduate years.

a In novices: current year of study. In intermediates: current year of residency. In experts: medical specialist since … years.

b Extended laparoscopic experience was determined as at least 50 performances for 2 of the selected procedures or more than 100 times for 1 of the selected procedures. Selected laparoscopic procedures are hysterectomy, oophorectomy, and pelvic lymphadenectomy in laparoscopic gynecology; cholecystectomy, Nissen fundoplication, bariatric procedures, and colectomy in laparoscopic surgery; and prostatectomy and nephrectomy in laparoscopic urology.

### Prior Experience

**Figure 2.**
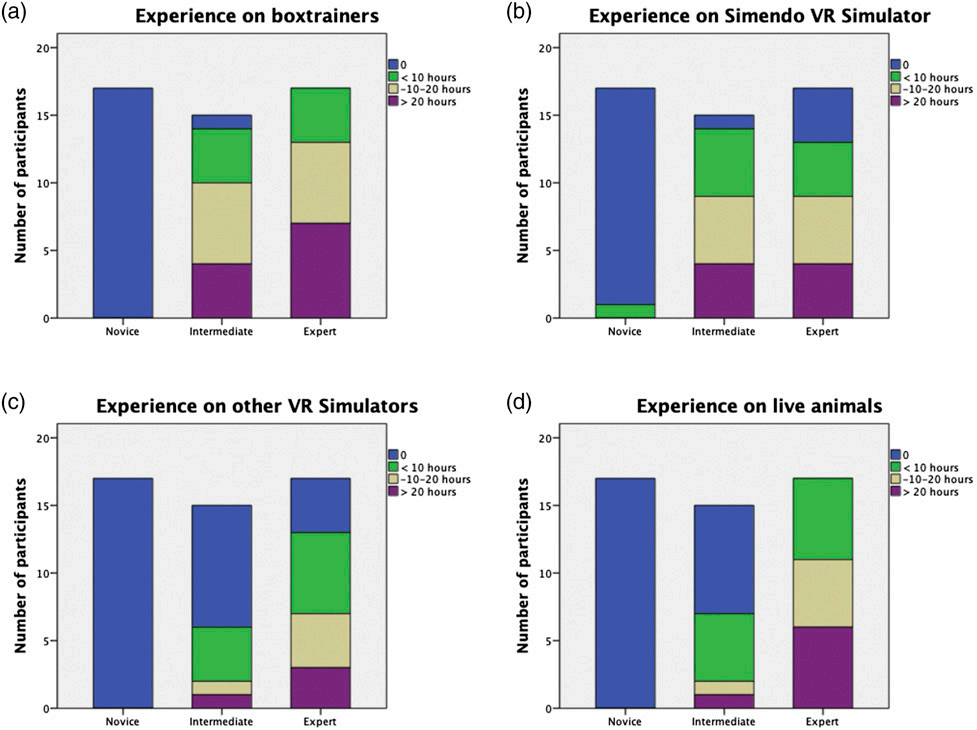
Prior experience on box trainers (A), Simendo VR simulator (B), other VR simulators (C), and live animal training (D) in hours.

None of the novices had prior experience on box trainers, other VR simulators, or live animal training. Only 1 of them had used the Simendo VR simulator before (during a one-hour session). Almost all intermediates had prior experience on box trainers (n = 14) and the Simendo VR simulator (n = 14). About half of the intermediates had experience on other VR simulators (n = 6) or live animal training (n = 7). All experts had experience on box trainers and live animal training, and the majority had experience on the Simendo VR simulator (n = 13) and other VR simulators (n = 13). In general, experts were most experienced, except for the Simendo VR simulator, where intermediates were most experienced. Prior experience for each group is demonstrated in Figure 2.

### Face Validity

The median scores considering the realism and training capacity of the curriculum are demonstrated in Table 3. The realism of the simulator was rated with median scores of 4.0 on all related questions except for interaction of the instruments with other objects and depth perception (median score 3.0). Questions regarding the training capacity of the curriculum were appreciated with median scores of 4.0, except for depth perception (median score 3.0). Concerning suitability of the curriculum to train PGY 1-3 residents, PGY 4-6 residents, consultants, and laparoscopic experts, median scores of, respectively, 4.0, 4.0, 4.0, and 3.0 were given. When comparing face validity rating scores between the 2 groups, there were no significant differences.

**Table 3.** Face Validity of the Bimanual Fundamentals Curriculum as a Whole.

Question	Group 2: Intermediate n = 15	Group 3: Expert n = 17	Overall n = 32	Significance Level^a^
What do you think of the realism of the curriculum concerning ...?				
(not realistic ... very realistic)				
Appearance of the instruments	4.0 (4.0-5.0)	4.0 (4.0-4.5)	4.0 (4.0-5.0)	.603
Movements of the instruments	4.0 (3.0-4.0)	4.0 (3.0-4.5)	4.0 (3.0-4.0)	.534
Freedom of movements of the instruments	4.0 (4.0-4.0)	4.0 (3.5-4.5)	4.0 (4.0-4.0)	1.000
Depth perception	3.0 (2.0-4.0)	3.0 (2.0-3.0)	3.0 (2.0-3.8)	.250
Interaction of the instruments with other objects	3.0 (3.0-4.0)	3.0 (2.5-4.0)	3.0 (3.0-4.0)	.765
What do you think of the training capacity of the curriculum ...?				
(very bad ... very good)				
In general	4.0 (4.0-4.0)	4.0 (4.0-5.0)	4.0 (4.0-4.0)	.702
Eye-hand coordination	4.0 (4.0-5.0)	4.0 (4.0-5.0)	4.0 (4.0-5.0)	.562
Depth perception	3.0 (3.0-4.0)	3.0 (2.0-3.5)	3.0 (2.0-4.0)	.237
Instrument navigation in general	4.0 (4.0-4.0)	4.0 (4.0-5.0)	4.0 (4.0-4.0)	.293
Training right and left hand separately	4.0 (4.0-5.0)	4.0 (4.0-4.5)	4.0 (4.0-5.0)	.497
Training cooperation between right and left hand (multiple tasking)	5.0 (4.0-5.0)	4.0 (4.0-5.0)	4.0 (4.0-5.0)	.102
The intermediate curriculum is suitable to train ...				
(not suitable ... very suitable)				
Residents PGY 1 to 3	4.0 (4.0-5.0)	4.0 (4.0-5.0)	4.0 (4.0-5.0)	.728
Residents PGY 4 to 6	4.0 (3.0-4.0)	4.0 (4.0-5.0)	4.0 (3.3-4.8)	.158
Consultants	4.0 (4.0-4.0)	4.0 (3.5-4.0)	4.0 (4.0-4.0)	.841
Laparoscopic experts	2.0 (2.0-3.0)	3.0 (1.5-4.0)	3.0 (2.0-3.0)	.251

Median scores (interquartile range) on a 5-point Likert scale. PGY = postgraduate years.

a *P* value for Kruskal-Wallis, nonparametric test.

Face validity scores per exercise are demonstrated in Table 4. Of all exercises, the training goal was reached (median scores of 4.0 for all exercises). The setup of the exercise, movement of instruments, and training capacity were rated with median scores of 4.0. Depth perception and the lack of haptic feedback were scored lowest (both scored 3.0 in the majority of the exercises). 96.9% agreed that implementation of the VR simulator was suitable in their current residency training programs. The other 3.1% answered “no opinion.” 71.9% felt the new Bimanual Fundamentals curriculum would be a good addition to the existing Simendo curricula. 28.1% answered with “no opinion.”

**Table 4.** Face Validity of the Individual Exercises of the Advanced Curriculum (n = 49).

Question	Sort the Rings	Stretch and Transfer	Ring and Rope	Balance	Puzzle
Do you think the training goal is reached?					
(Not at all ... yes for sure)	4.0 (4.0-4.8)	4.0 (4.0-5.0)	4.0 (4.0-4.0)	4.0 (4.0-5.0)	4.0 (3.0-5.0)
What do you think of …?					
(Very bad … very good)					
Setup of the exercise	4.0 (4.0-5.0)	4.0 (4.0-4.8)	4.0 (4.0-5.0)	4.0 (4.0-5.0)	4.0 (3.0-5.0)
Movements of the instruments	4.0 (4.0-4.0)	4.0 (4.0-4.0)	4.0 (3.3-4.0)	4.0 (3.0-4.0)	4.0 (3.0-4.0)
Depth perception	3.0 (3.0-4.0)	4.0 (3.0-4.0)	3.0 (2.0-4.0)	3.0 (3.0-4.0)	3.0 (2.0-4.0)
Trainings capacity of the exercise	4.0 (4.0-4.0)	4.0 (4.0-4.0)	4.0 (4.0-5.0)	4.0 (3.5-4.0)	4.0 (3.0-4.0)
Lack of haptic feedback (very disturbing ... not disturbing at all)	3.0 (2.0-4.0)	3.0 (2.0-4.0)	3.0 (2.3-4.0)	3.0 (2.0-4.0)	3.0 (2.0-4.0)

Median scores (interquartile range) on a 5-point Likert scale.

### Construct Validity

Median task time, collisions/displacements, and path length right and left scores of the second and third run are presented in Table 5. The comparison between the novices and the other cohorts showed the most significant differences. The parameter task time was significantly different in all 5 exercises between groups (*P* < .001 for all 5 exercises). Both experts and intermediates outperformed the novices in all exercises (Table 6). In addition, the expert group performed 2 exercises quicker than the intermediate group: “*Ring and Rope*” time 106.51 (90.09-118.65) vs 132.94 (103.99-163.37) *P* = .024 and “*Puzzle*” time 163.62 (152.89-195.74) vs 213.70 (176.31-242.64) *P* = .004.

**Table 5.** Construct Validity of the New Advanced Curriculum (n =49).

	Novice n = 17	Intermediate n = 15	Expert n = 17	Significance Level^a^
Sort the rings				
Task time	212.70 (176.43-241.96)	136.11 (128.40-150.34)	138.89 (128.40-151.01)	<.001
Collisions	8.50 (5.25-15.00)	7.50 (5.00-10.50)	3.00 (1.50-7.00)	.002
Path length R	252.00 (217.75-313.50)	193.50 (161.00-215.50)	178.50 (165.25-190.25)	<.001
Path length L	291.50 (223.25-350.75)	202.00 (172.50-222.50)	185.50 (172.00-220.00)	<.001
Stretch and transfer				
Task time	254.07 (223.62-294.54)	127.50 (113.68-176.29)	124.22 (108.26-149.51)	<.001
Collisions	8.50 (6.25-13.00)	5.00 (2.50-5.50)	4.00 (3.00-7.25)	.006
Path length R	237.00 (214.50-284.50)	162.50 (150.50-175.00)	172.00 (166.00-190.00)	<.001
Path length L	231.00 (207.75-270.50)	181.00 (157.50-187.00)	174.50 (154.25-183.75)	<.001
Ring and rope				
Task time	273.99 (187.56-344.77)	132.94 (103.99-163.37)	106.51 (90.09-118.65)	<.001
Collisions	4.00 (1.00-6.75)	1.50 (1.00-3.00)	1.00 (.50-1.75)	.009
Path length R	203.00 (142.50-275.75)	125.00 (98.00-179.00)	120.50 (106.00-134.00)	.001
Path length L	264.50 (182.75-338.75)	150.00 (124.00-183.50)	139.00 (112.00-164.25)	<.001
Balance				
Task time	183.80 (164.89-238.22)	149.27 (112.26-174.23)	125.91 (119.33-137.86)	<.001
Displacement	.50 (.00-1.25)	.50 (.00-1.00)	.50 (.00-1.25)	.856
Path length R	143.50 (113.00-171.00)	116.50 (97.00-145.50)	101.50 (89.25-128.00)	.006
Path length L	121.50 (111.25-150.75)	92.50 (76.00-111.00)	83.00 (75.00-95.00)	<.001
Puzzle				
Task time	381.64 (301.48-443.01)	213.70 (176.31-242.64)	163.62 (152.89-195.74)	<.001
Path length R	479.50 (412.25-734.25)	283.00 (220.00-424.50)	242.50 (182.75-280.25)	<.001
Path length L	447.50 (382.25-550.25)	287.50 (255.50-354.50)	199.00 (177.25-228.00)	<.001

Median scores (interquartile range), Abbreviations: R = right, L = left.

Task time in seconds; path length, collisions, and displacement in arbitrary units.

a *P* value for Kruskal-Wallis, nonparametric test.

**Table 6.** Construct Validity: Significance Levels Between Groups (n = 49).

	Novice vs Intermediate	Novice vs Expert	Intermediate vs Expert
Sort the rings			
Task time	<.001	<.001	.794
Collisions	.390	.001	.009
Path length R	.001	<.001	.313
Path length L	<.001	<.001	.602
Stretch and transfer			
Task time	<.001	<.001	.551
Collisions	.004	.008	.794
Path length R	<.001	<.001	.082
Path length L	<.001	<.001	.350
Ring and rope			
Task time	<.001	<.001	.024
Collisions	.033	.003	.331
Path length R	.005	<.001	.576
Path length L	.001	<.001	.123
Balance			
Task time	.004	<.001	.350
Displacements	.682	.658	.911
Path length R	.069	.001	.176
Path length L	.004	<.001	.390
Puzzle			
Task time	<.001	<.001	.004
Path length R	<.001	<.001	.049
Path length L	<.001	<.001	.001

*P* values (Mann-Whitney U nonparametric test). Abbreviations: R = right, L = left.

The parameter collisions/displacements were measured in 4 of 5 exercises. It was not a relevant parameter in the “*Puzzle*” exercise. There were significant differences between groups in 3 of 4 exercises. In the exercise “*Sort the Rings*,” the experts made significantly fewer collisions than the novices (3.00 (1.50-7.00) vs 8.50 (5.25-15.00) *P* = .001) and intermediates (3.00 (1.50-7.00) vs 7.50 (5.00-10.50) *P* = .009). In the exercise “*Stretch and Transfer*,” both experts and intermediates outperformed the novices with a significant difference (4.00 (3.00-7.25) vs 8.50 (6.25-13.00) *P* = .008, 5.00 (2.50-5.50) vs 8.50 (6.25-13.00) *P* = .004, respectively). Also, in the exercise “*Ring and Rope*,” the experts and intermediates made significantly fewer collisions than the novices (1.00 (.50-1.75) vs 4.00 (1.00-6.75) *P* = .003, 1.50 (1.00-3.00) vs 4.00 (1.00-6.75) *P* = .033, respectively). In the exercise “*Balance*,” the parameter displacements were acquired, which showed no significant differences between groups.

Path length was obtained for both right and left instrument separately. Experts and intermediates significantly outperformed novices in all 5 exercises (Tables 5 and 6). When comparing experts with intermediates, a statistically significant difference was found for both path length left and path length right in the exercise “*Puzzle*” (242.50 (182.75-280.25) vs 283.00 (220.00-424.50) *P* = .049, 199.00 (177.25-228.00) vs 287.50 (255.50-354.50) *P* = .004, respectively). Moreover, in exercises “*Ring and Rope*” and “*Balance*,” a trend was found in favor of the experts (*P* = .123 and *P* = .176, respectively).

Median total performance scores of each group and their significance levels can be found in Table 7. Both experts and intermediates scored higher than novices (*P* < .001 and *P* < .001, respectively). Experts also outperformed intermediates, but the difference was too little to be statistically significant (*P* = .153).

**Table 7.** Total Performance Score with Significance Levels (n = 49).

	Novice^a^n = 17	Intermediate^a^ n = 15	Expert^a^ n = 17	Novice vs Intermediate^b^	Novice vs Expert^b^	Intermediate vs Expert^b^
Total performance score	26.0 (22.5-34.0)	43.0 (34.0-47.0)	45.0 (41.0-49.5)	<.001	<.001	.153

a Median (interquartile range).

b *P* values (Mann–Whitney U nonparametric test).

## Discussion

### Main Findings

The purpose of this study was to determine face and construct validity for the new “*Bimanual Fundamentals*” curriculum for this VR simulator. We demonstrated face validity with overall positive scores. A Likert score of 4 out of 5 has been reported as an adequate score to demonstrate face validity, while a score of 3 out of 5 has been reported as acceptable.^24^ Both realism and training capacity of the simulator scored 4 out of 5 on all items, except for depth perception which scored a 3. The individual exercises were also rated high on the majority of the questions. The lack of haptic feedback scored the lowest, with a median score of 3 out of 5. Despite the lower scores on depth perception and lack of haptic feedback, almost all of the intermediates and experts felt the new curriculum is a good addition to the existing curricula and suitable as a training tool in their residency programs.

We were able to establish decent construct validity by demonstrating statistically significant differences between performers with different levels of experience. In all 5 exercises, experts outperformed novices on the great majority of the measured parameters. In 2 exercises, the experts also outperformed the intermediates on various parameters. This was mainly the case in the more difficult exercises (ie., “*Ring and Rope*” *and* “*Puzzle*”), where intensive cooperation between the right and left instruments was crucial. Experts were not only faster but also had a better economy of movement. In the exercise “*Sort the Rings*,” experts made significantly fewer collisions than the other groups. This is in line with the thought that experts are highly efficient in their movements and when performing a less challenging exercise, are better able to optimize the execution.

### Explanation of Main Findings

Due to their relatively low scores, depth perception, the lack of haptic feedback, and interaction of the instruments with other objects were indicated as limitations of this particular simulator. Depth perception and interaction with objects are harder to achieve on a 2D interface and are a limitation in both VR simulating interfaces and real laparoscopic surgery.^25^ During the development of software for this simulator, clear shadows and the use of different colors for different objects were added in order to improve both depth perception and interaction with objects. Nonetheless, participants rated both as mediocre. This might have been partly caused by the relatively small display that was used. A bigger display may further enhance depth perception.

The lack of haptic feedback is often stated as a major disadvantage of VR simulators in comparison with box trainers or training on live animals or cadavers. In this study, the participants experienced the lack of haptic feedback as moderately disturbing. Opinions and literature about haptic feedback in VR simulators are ambiguous. Some surgeons believe that haptic feedback is an important part of a VR simulator,^26^ while others indicate that simulation outcome for exercises augmented with haptic feedback is likely to be inaccurate, resulting in a not better or even negative training effect.^27,28^ Recent studies found none or minor improvements in training effect using haptic feedback.^10,29,30^ Adding realistic haptic feedback is difficult, and it is usually an expensive add-on to VR simulators.^31^ The evidence for transfer of skills using nonhaptic feedback VR simulators is well established.^9,10,14-17^ Therefore, haptic feedback seems, in the current technical state, not a cost-effective feature in VR simulators for minimally invasive surgery.

Regarding construct validity, we found that the parameters task time, collisions, and path length right and left are valuable parameters for performance assessment on this particular simulator because of the significant differences between groups. These metrics have also been validated in previous studies.^32-34^ The parameter displacements were only measured in the exercise “*Balance*” and may be less suitable to assess performance, since the simulator was not able to differentiate between a novice and an experienced user. The number of displacements was very low in all groups. A displacement was registered when a weight was put on the scale, while the scale was not balanced, whereas the main goal of the exercise was to put the weights on a balanced scale. Other “mistakes,” like dropping a weight off the scale or hit the scale with 1 of the instruments, were not recorded. Presumably, participants were mainly focusing on putting weights on a balanced scale and therefore made none to little displacements, but more often they made other mistakes. Another form of error measurement might be more accurate and suitable to assess performance in this exercise.

It has been a point of discussion in previous studies that parameters (especially task time) on its own do not provide enough information to distinguish different levels of expertise.^9,33^ Therefore, we calculated an objective total performance score to assess participants’ performance with all measured parameters combined. As expected, both experts and intermediates achieved significantly higher scores than novices. Moreover, experts scored higher than intermediates, but the difference was too little to be statistically significant.

Overall, the discriminative abilities of the curriculum between experts and intermediates seemed small. This can be interpreted in several ways. First, the intermediate group consisted of senior residents only, advanced in their residency program (PGY 4-6). This designates a moderate to high level of laparoscopic experience. Second, the curriculum was developed for advanced laparoscopic skills training, whereas the current state of assessment systems is not able to adequately distinguish between levels of higher skill.^35^ This finding is therefore not really unexpected and consistent with other studies aiming to validate simulators as training tools.^36-38^ Third, it can be argued that participants with previous experience on this specific simulator would perform better than others. To investigate if prior experience influenced a participants’ total performance score, a correlation analysis was performed. We indeed found a correlation of 17.6% (*r*_*s*_ = .419, *P* = .017), which confirms the hypothesis that prior experience will lead to a better performance. Since experience on this particular simulator was higher in the intermediate group than in the expert group (93.3% of the intermediates vs 76.5% of the experts had experience on the Simendo VR simulator), we conclude that prior experience on this simulator can partly explain the relatively high performance of intermediates in comparison with the expert group.

### Strengths and Limitations

A strength that should be mentioned is that we calculated a total performance score for each participant. This gave us the chance to compare participants’ total performance, instead of only comparing a single parameter. In real practice, technical expertise is also dependent on a combination of performing time efficiently, with economy of movement and without making mistakes.

Besides the unequal distribution of prior experience among groups, some other limitations should be noted. First, the relatively small sample size could be indicated as a limitation, but we performed a power analysis and reached a sufficient sample size. Moreover, our study was conducted at multiple medical centers. Therefore, we believe the relatively small sample size did not influence the generalizability of our results substantially.

Second, participants needed between 1 and 1.5 hours to complete the 5 consecutive exercises without a pause. Considering the increasing level of difficulty with each following exercise, this requires persisting concentration and perseverance. It may be possible that participants lost their focus or interest and did not perform at their best, especially in the last exercises. Since only a small number of participants complained about this, we believe this did not influence the results significantly.

Third, the setup of the simulator could have been more optimal. As stated before, the laptop that was used had a 15.4-inch display, which is relatively small. Also, it was not possible to adjust the height of the simulator instruments. For some participants, this could have been working to their disadvantage. This could also be seen as a strength since we maintained a controlled and identical height for every participant.

Fourth, there was only a significant difference between the intermediate and expert group in 2 exercises. An explanation could be that the intermediate group had more prior experience on this simulator (Figure 2.)

### Future Research

Future research can determine whether prior experience on this simulator moderates the relationship between surgical expertise and performance on this VR simulator. Also, it would be interesting to add a PGY 1-3 group to the sample to further determine the simulators’ abilities to differentiate between moderate to high levels of skill.

## Conclusion

We determined face and construct validity for the new “*Bimanual Fundamentals*” curriculum for this particular simulator. Overall, reality was rated high, and the training goals of each exercise were reached. The simulator is well able to differentiate between performers’ experience levels, and we therefore believe that this curriculum is useful as a training and assessment tool for residents’ psychomotor skills. We opt to integrate VR simulator training curricula in residents’ training curricula, where residents have to reach a certain performance level on the VR simulator before being allowed to perform laparoscopic procedures in real practice. It is therefore important to use validated exercises only, preferably with different levels of difficulty, so residents will be assessed objectively. Using exercises with different levels of difficulty will also keep residents motivated to practice more and therefore, finally, improve surgical outcomes.
